# Effects of a Multi-Component Training Program on Healthy Older Adults' Prospective Memory Performance: Assessing Change Over Time

**DOI:** 10.3389/fpubh.2021.594953

**Published:** 2021-04-22

**Authors:** Azin Farzin, Rahimah Ibrahim, Zainal Madon, Hamidon Basri, Shervin Farzin, Abbas Motalebizadeh

**Affiliations:** ^1^Malaysian Research Institute on Aging, Universiti Putra Malaysia, Serdang, Malaysia; ^2^Department of Clinical Psychology, University of Social Welfare and Rehabilitation Sciences, Tehran, Iran; ^3^Department of Human Development and Family Studies, Faculty of Human Ecology, Universiti Putra Malaysia, Serdang, Malaysia; ^4^Department of Medicine, Faculty of Medicine and Health Sciences, University Putra Malaysia, Serdang, Malaysia; ^5^Faculty of Information System, University Technology Malaysia, Johor Bahru, Malaysia; ^6^Department of Biomedical Engineering, School of Mechanical Engineering, Iran University of Science and Technology, Tehran, Iran

**Keywords:** prospective memory, multi-component, training program, older adults, training duration

## Abstract

Prospective Memory (PM) is a cognitive function affected by aging. PM is the memory of future intentions and is significantly involved in everyday life, especially among older adults. Nevertheless, there are a few studies focused on PM training among healthy older adults and these studies did not report the optimal duration of training regarding improving PM performance among older adults. The present study aimed to determine the effective duration for training PM performance among healthy older adults. The current study was a randomized, controlled, single-blind, within-participants crossover trial including a training program with a duration of 12 h. The sample of 25 older adults aged 55 to 74 years recruited from the active members of the University of the Third Age (U3A), Kuala Lumpur/Selangor, their family members, and friends. The study design ensured some participants would receive the training after baseline while others would wait for 6 weeks after the baseline before receiving the training. All participants were evaluated five times: at baseline, 6, 12, 16, and at 24 weeks post-baseline. Moreover, the training program ensured all participants were assessed after each training session. The minimum number of hours to achieve training effects for this multi-component training program was eight. Results supported the efficacy of the training program in improving PM performance among healthy older adults. Also, the optimal duration for the multicomponent training program on PM performance among healthy older adults was obtained. This trial is registered at isrctn.com (#ISRCTN57600070).

## Introduction

Improving life expectancy and the age-related cognitive changes encourage researchers to develop new training approaches to promote healthy aging, independent living and prevent cognitive declines in older adults (1, 2). There is a global interest in cognitive training for older adults and most cognitive training studies demonstrated promising results (3). Strategy-based cognitive trainings improved specific cognitive functions in a compensatory manner, whereas process-based cognitive trainings used an intensive restorative manner to promote a specific cognitive function by exercising the underlying cognitive mechanisms of it repeatedly (4). Previous cognitive trainings primarily focused on specific populations (i.e., clinical), cognitive functions (i.e., working memory) or used a single training approach (i.e., strategy-based). Despite great benefits of strategy- or process-based trainings as independent approaches, to accomplish optimal results, there is an urgent need to use a combination of these two approaches (i.e., multi-component) across a greater range of cognitive functions among healthy older adults' population (5–8).

One of the most suitable targets for a multi-component training program is prospective memory (PM) which is a relatively neglected cognitive function in memory field of research. PM seems to be trivial, but has an important role in regard to having a successful and independent everyday life, especially among older adults (6, 9). PM is involved in remembering to perform an intended action in future (9). It is the memory for daily living tasks, including self-care (e.g., medication adherence), grocery shopping, cooking, and keeping appointments. PM is a critical cognitive function for older adults as it can promote self-care, independence, and well-being among them (9, 10). Wherefore, it is significant to maintain and promote PM performance among older adults.

Based on the nature of the “cue,” there are three types of PM, including time-based, event-based, and activity-based PM (11). PM is a multi-phase cognitive process with four phases: (i) intention formation, (ii) delay maintenance interval, (iii) self-initiated cue recognition and intention retrieval, and (iv) intention execution (12). Being a multi-phase and multi-process cognitive function and closely associated with executive functions, attention, planning and several other cognitive functions, made PM a suitable target for a multi-component cognitive training (i.e., strategy- and process-based training) which would simultaneously target several cognitive functions including attention, cognitive control, memory, reasoning, and executive functions in one training program (12). Such holistic training program can show significant effects on all aspects of older adults' real life and well-being (6).

In addition, to boost the memory training effects, the training should be cost-effective, acceptable and tailored. Such program can incorporate older adults' personal differences and resources efficaciously (13, 14). Moreover, stronger experimental designs should be developed for such studies to show accurate results (15). However, there are a few studies aimed to evaluate or improve PM performance among older adults (16, 17), and there are fewer studies aimed to promote PM performance among healthy older adults (18–20).

Consequently, the primary study was designed to be a crossover trial and evaluated the effectiveness of a tailor-made preventative multi-component training program among healthy older adults (21). In that study, the multi-component training program including strategy- (e.g., implementation intentions) and process-based (i.e., Virtual Week (VW) computer-based board game) components was conducted on a group of healthy older adults to train their PM performance. The results showed that besides improving older adults' PM performance, training PM performance can cause older adults' levels of anxiety and negative mood (i.e., psychological well-being primary factors) to decrease and their level of independence was increased. Moreover, the effects of the training were persistent after 3 months from the last intervention session (21).

The VW board game simulates a number of real-life PM tasks to train time- and event-based PM performance. The VW paradigm has a general storyline for seven virtual days and each day includes an individual story with a number of PM tasks to perform (22, 23). The paradigm allows the examiner to record the performance of the participants for each training session. Hence, as the training program provided room to evaluate the participants' PM performance at the end of each session, for the current study, the optimal training duration was aimed to be found to aid future studies in regard to planning more cost-effective training programs for older adults (21).

## Methodology

The current study was a randomized, controlled, single-blind, within-participants crossover trial with 4- and 12-week follow-ups. The CONSORT statement was followed as the main framework to develop the methodology of the current study (24). After the baseline assessments were conducted, participants were randomly assigned into the treatment or control conditions. A 6-week tailored multi-component PM training program was conducted for the treatment group. The training program was consisted of two different components: process-based, and strategy-based components. The participants in control condition were not contacted during the training phase. After 6 weeks, participants crossed over and underwent the condition they had not experienced before.

### Participants

Participants were recruited from a pool of active members of the University of the Third Age (U3A) association Kuala Lumpur/Selangor, and the invitation to participate in the study was extended to their family members, and friends. A total number of 31 participants were screened before the group allocation. However, not all of them were able to participate in the study, and only 25 out of 31 joined the program. There were 6 men and 19 women Age ranged from 55 to 74 years.

There is a need to define the concept of “older adult” which could be defined in various ways. Based on the (25) definition, while in numerous (westernized) countries, older adults are defined as individuals who are 60 years of age and above, various issues must be considered to define the concept of “older adults” in different countries. Explicitly, older adults should not be solely described according to the chronological age (25). Correspondingly, (18) proposed that several factors including abilities, resources, and the training target (e.g., PM performance) should be considered to describe “older adults” (18). Due to the fact that the nature of the primary and the current study and based on the World Health Organization strategies, older adults were described in accordance with the age of retirement from a paid job (i.e., receiving a retirement pension) (25). The retirement age for Malaysians had been changed from 55 to 60 years in 2013, and the study cohort for this study was at least 56 years old in that year, hence they retired at the age of 55. Therefore, the lower age range for the current study was age of 55.

Furthermore, because there are several factors involving in older adults' training, it is not unconventional to view people who are 55 years old and above as older adults (18). The target for the primary study was PM performance and the previous studies illustrated the changes associated with age in PM performance can be noticed in early stages of old age (9, 20, 26, 27). Accordingly, the ideal age to have preventative PM performance training programs for older adults was proposed to be 55 years and above (18, 20).

Additionally, based on the literature, older adults' age range is not a fixed factor and numerous studies considered various age ranges as older adults in line with their aim and study target (18, 28–31). Reviewing the literature, in most studies about older adults, young and old elders were both viewed as “older adults” which could be a concern about the equal benefits of cognitive training for these age groups. However, both age groups may gain equally from cognitive training programs (3, 32) due to the fact that the ability to learn remains mostly intact even in very old adults (33).

Eventually, the upper age limit for the primary study was considered based on the study location, the inclusion criteria of the study (21) and the participants' characteristics and some of the most important ones were: (i) one of the most significant inclusion criteria for the primary study was good command of English (because the program was conducted in English) and due to the fact that partaking in this training program required a minimum level of education, at least the secondary level of education was needed for the potential participants to be enrolled in the training program. Nonetheless, not many Malaysian elders have higher levels of education and a good command of English (34), and (ii) the risk of having (at least) mild cognitive impairments among Malaysian elders who are 75 years old and above is high (34).

Participants were screened on a single occasion prior to the baseline assessments to ensure they met the inclusion criteria of the study which included the absence of: (i) any neurological impairments, assessed with Mini-Mental State Examination (MMSE), (ii) any chief psychiatric disorders and learning disabilities, (iii) having experienced head traumas, general anesthesia, or cerebrovascular disease, and (iv) drug/alcohol abuse. Moreover, the lower educational level among participants was at least secondary educational level and they were required to have a good command of English. All participants were informed about the nature of the study and provided informed consent forms prior to entering the study.

### Baseline Measurements

All measurements used in this study showed high reliability and validity in clinical or older adults' populations and they are as follows: (i) Geriatric Depression Scale (GDS): a 30-item self-reported questionnaire used to measure the depression level among participants (35), (ii) Geriatric Anxiety Scale (GAS): a 30-item self-reported scale used to assess anxiety symptoms among older adults (36), (iii) Instrumental Activities of Daily living Scale (IADL): a measurement used to evaluate daily living activities (37), (iv) Prospective and Retrospective Memory Questionnaire (PRMQ): a 16-item questionnaire to assess the subjective retrospective (RM) and PM performance (38), and (v) Prospective Memory Tasks; a computerized program which was adapted from a similar program used in other similar studies to assess the objective PM performance (39).

### The Intervention Phase

The intervention included a 12-h (1 session per week, 2 h per-session) PM training program. It consisted of two main parts including process-, and strategy-based components. For the strategy-based component, “implementation intentions” (40) and instructions were used regarding participants' everyday PM tasks (e.g., self-care, social appointments). Participants wrote down a list of their daily living activities and how they usually performed them (e.g., monitoring their health, keeping appointments, grocery shopping). Next, the participants were instructed to transform the information to PM tasks through implementation intentions strategy (e.g., If I am home next Friday at 6.30 pm, I will call my friend and ask for her measuring cup). Furthermore, they were asked to visualize all necessary steps to fulfill a task. The process-based component included VW Board Game (22, 23). This game is a computerized board game in which participants move their tokens around the board with the roll of a dice. Each circuit of the board represents one virtual day, and the game includes 7 virtual days (and 1 trial day). Participants should make choices about different daily activities (ongoing tasks) and remember to do some other activities (PM tasks). A “perform task” button is placed on the game screen and participants ought to open it to select the task to perform. Each day of the VW game includes four regular, four irregular, and two stop clock tasks. The regular PM tasks represent usual daily PM tasks, such as taking blood pressure medication (two time-based, and two event-based tasks). The four irregular PM tasks are similar to occasional PM tasks which may occur in everyday life. These tasks also include 2 Time-based, and two event-based tasks (e.g., inviting a friend for dinner). There are two stop clock tasks, as well. Answers on VW are scored as follows: (i) correct: if the token is moved to or past the target square (on the board) immediately after the roll of dice for that task and before the next roll of the dice (for stop clock tasks, correct is to complete the task at the target time on time or within next 10 sec), (ii) little late: completing the task after the correct time passed but before next event card (for event-based), 1 h, and 30 sec passed for time-based, and stop clock tasks, (iii) late: conducting the task after little late condition and before the end of that virtual day, (iv) little early: completing the task before the correct answer criterion and after the little late answer criterion for the previous event card, 1 h, and 30 sec for event-based, time-based, and stop clock tasks, respectively, (v) early: performing the task before the little early answer criterion and after the start of that virtual day, (vi) missed: the task was not performed at any time, (vii) cancel: when one opens the perform task list and closes it without selecting a task, and (viii) wrong: when a distractor task (there are some distractor tasks listed with PM tasks in the task list of each day) is selected. The game has a high reliability and consistency (22, 23, 39). For the current study, task difficulty was increased successively by increasing the number of tasks and hiding the clock from the screen so, time monitoring would be more complex. The game is designed to calculate and reveal participants results for each virtual day which provided a possibility to evaluate the change in participants' performance over the time. The results from VW were recorded for all participants after each session to be analyzed (measuring the change in their performance during the study period).

### Outcome Measures

Primary outcome measures of the current article were PM functions (time-, and event-based PM) measured with VW. The secondary outcome measures included the levels of independence, and psychological well-being (i.e., anxiety, depression).

### Statistical Analysis

All sessions results reported as means (M), and standard deviations (SD). The General Linear Model (GLM) was utilized to show change over time. The level of significance was set as *p* < 0.05. Moreover, analyzing the data on an intention-to-treat basis was considered.

## Results

Although 31 participants were assessed initially for eligibility to participate in the current study, 25 participants entered the study eventually and they were randomly assigned into either the training or control groups. The groups did not show any significant differences in regard to their demographics and baseline characteristics (Table 1). All participants attended all training, and follow-up sessions.

**Table 1:** Study cohort demographics and baseline measures.

	**Study cohort (*n* = 25)**	**Treatment group (*n* = 13)**	**Control group (*n* = 12)**	***P-*Value**
Number	25	13	12	
Age (Range)	55–74	55–74	55–71	
Mean age (years)	63.32 ± 4.44	63.69 ± 4.83	62.92 ± 4.14	t = 0.79
Women/Men	19/6	10/3	9/3	χ 2 = 0.81
Years of Education (Range)	10–20	10–20	11–20	
Mean years of education	14.04 ± 3.07	14.69 ± 3.37	13.33 ± 2.67	t = 0.61
Cognitive State (Range)	27–29	27–29	27–29	
Mean Cognitive State	27.68 ± 0.74	27.62 ± 0.76	27.75 ± 0.75	t = 0.66
**PM Tasks**				
Time-based PM		0.77 ± 1.36	0.92 ± 1.08	*P* > 0.05
Event-based PM		3.23 ± 1.64	2.33 ± 1.37	*P* > 0.05
Activity-based PM		1.31 ± 0.94	1.17 ± 0.71	*P* > 0.05
**PRMQ**				
PM		28.85 ± 3.41	27.33 ± 2.93	*P* > 0.05
Total		54.62 ± 6.00	50.92 ± 3.91	*P* > 0.05
IADL		6.38 ± 0.65	6.08 ± 0.99	*P* > 0.05
GDS		7.46 ± 1.33	5.75 ± 1.13	*P* > 0.05
GAS		19.08 ± 2.66	19.8 ± 2.19	*P* > 0.05

**Figure 1:**
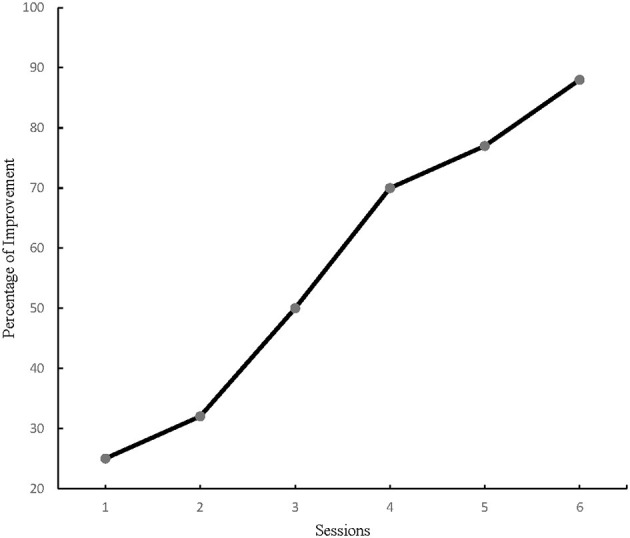
Time-based PM performance change over time.

**Figure 2:**
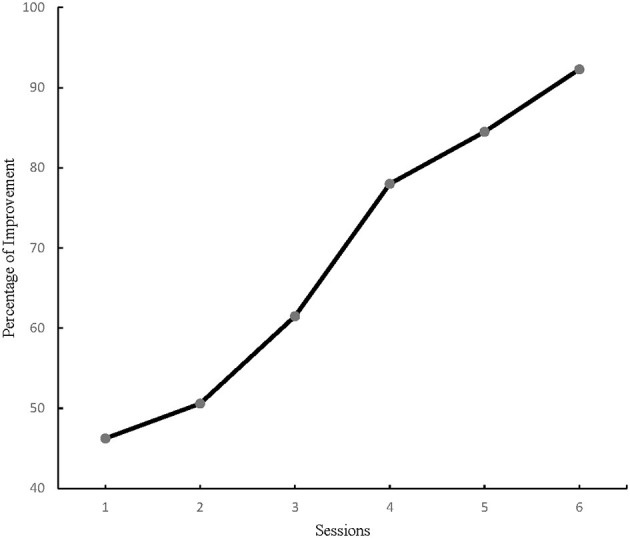
Event-based PM performance change over time.

The number of PM tasks increased continuously during the intervention phase therefore, at the end of each session, the percentage of correct answers were recorded for each participant. The primary outcome for the current study was PM performance measured with VW. The results for time-based PM performance analysis demonstrated a significant difference for within-subjects effect from the first to the last training session in the study cohort, *F* (5, 144) = 39.38, *p* = 0.00, η^2^ = 0.57. And the repeated GLM analysis of time-based PM performance showed a significant change between the third and the fourth sessions (Figure 1), *F* (1, 48) = 8.70, *p* < 0.005, η2 = 0.15. Consequently, all participants' time-based PM performance improved considerably by the fourth training session (Table 2). Similarly, event-based PM performance results showed a significant difference from the first to the last training session among all participants *F* (5, 144) = 17.41, *p* = 0.00, η^2^ = 0.37. Moreover, the repeated GLM analysis of event-based PM performance illustrated a significant change between the third and the fourth sessions (Figure 2), *F* (1, 48) = 4.66, *p* < 0.005, η2 = 0.08. Accordingly, all participants' event-based PM performance improved significantly by the fourth training session (Table 3). In sum, the results from this study suggested the minimum training duration to improve PM performance among healthy older adults was 8 h.

**Table 2:** The VW game results for within-subject effects on time-based PM performance.

	**M ± SD**			***F* (*p*)**			
**Session**		**1^**st**^ Sess**.	**2^**nd**^ Sess**.	**3^**rd**^ Sess**.	**4^**th**^ Sess**.	**5^**th**^ Sess**.	**6^**th**^ Sess**.
1^st^ Sess.	46.00 ± 21.26		00.54 (*p* > 0.05)				
2^nd^ Sess.	75.00 ± 50.50			1.89 (*p* > 0.05)			
3^rd^ Sess.	60.99 ± 31.24				4.66 (*p* < 0.05)		
4^th^ Sess.	77.50 ± 21.94					1.12 (*p* > 0.05)	
5^th^ Sess.	84.20 ± 22.62						2.26 (*p* > 0.05)
6^th^ Sess.	91.85 ± 11.59						

*Sess, Session; M, mean; SD, standard deviation*.

**Table 3:** The VW game results for within-subject effects on event-based PM performance.

	**M ± SD**			**F (*p*)**			
**Session**		**1^**st**^ Sess**.	**2^**nd**^ Sess**.	**3^**rd**^ Sess**.	**4^**th**^ Sess**.	**5^**th**^ Sess**.	**6^**th**^ Sess**.
1^st^ Sess.	23.32 ± 8.33		3.15 (*p* < 0.05)				
2^nd^ Sess.	30.99 ± 19.91			6.90 (*p* > 0.05)			
3^rd^ Sess.	48.88 ± 27.59				8.70 (*p* < 0.05)		
4^th^ Sess.	68.99 ± 20.02					1.35 (*p* > 0.05)	
5^th^ Sess.	76.26 ± 24.04						2.70 (*p* > 0.05)
6^th^ Sess.	85.61 ± 15.18						

## Discussion

As it was mentioned, PM is a significant cognitive function in regard to older adults' everyday life. Nevertheless, the body of literature regarding PM training programs among older adults is a very limited one (6) and there is a limited number of studies focused on the effectiveness of the training programs in terms of time and energy needed for such programs (13, 14). Likewise, there are a few studies aimed to improve PM using either strategy- or process-based approaches (5, 8, 19) and they showed some limitations; the primary focus of the process-based approaches were on the working memory (6, 19) and most strategy-based training programs either aimed to maintain or improve PM performance in a clinical population or they focused on just one everyday task (e.g., health tasks) (5, 7, 8). Either of these training approaches were shown to be effective; however, they did not show any significant and/or persistent effects. Incorporating strategy-based techniques in the current training program boosted the efficiency of the process-based techniques and showed to have significant training effects which was in line with the previous studies results (7, 18). Moreover, by combining strategy-, and process-based techniques, this study exceeded some limitations of the previous studies (6, 18–20). In addition, being “cost-effective” is one of the most significant characteristics of a training program, especially for older adults because there are several factors which may have large negative impacts on the results of the program (e.g., the increased number of drop-outs) (41). For instance, while this study had an excellent retention rate, one of the important drawbacks of this study was the number of participants. A number of individuals did not partake in the current training program due to reasons including transport costs, length, and time schedule of the program. Therefore, following the results of some previous studies (13), this study investigated the optimal training duration for a multi-component PM training program.

Being able to deliver a program which would address older adults' needs in an effective manner should be considered as a key factor to design a training program for these individuals. Cost-effectiveness as a concept could be defined as a characteristic of a training program which allows the program to have the optimal effects utilizing the least number of resources (e.g., time, energy, money). Hence, computing the least number of resources and taking them into consideration before designing and conducting a program seems to be crucial (13, 14).

Results from VW revealed all participants made significant gains from the training program. They demonstrated to have improvement in their PM performance from the first intervention session to the last one. However, the significant change in their performance was achieved by the end of the fourth training session. Although participants kept improving after 8 h of training, their improvements were not significant. Therefore, this study found 8 h of a tailor-made multi-component training program is sufficient to improve PM performance among healthy older adults. These results could aid future similar studies to save time, energy and effort regarding developing and conducting similar programs (13, 21).

Although these results are encouraging, there were some limitations faced during this study. As it was mentioned before, the sample size in this study was rather small which led to some small yet potentially significant results. Nevertheless, the current study findings suggest further examination in a larger sample to extend the benefits of PM training among older adults. Another important limitation of the current study was not having long-term follow-ups (e.g., 12 months) to be able to show any possible long-term training effects. Furthermore, as the current study combined strategy-, and process-based approaches, the training gains of each approach remained unclear. The efficacy (i.e., training and cost effects) and generalizability of these two approaches are significant issues to investigate in future studies.

## Conclusion

Partaking in a customized multi-component PM training program resulted in significant training effects in PM performance. Training gains led to near and far transfer effects in the form of improved levels of independency and well-being. Additionally, this study was able to assess and achieve the optimized effective training duration to encourage future studies to design and conduct more cost-efficient training programs.

## Data Availability Statement

The raw data supporting the conclusions of this article will be made available by the authors, without undue reservation.

## Ethics Statement

The studies involving human participants were reviewed and approved by The Ethics Committee for research involving human subjects of University Putra Malaysia (JKEUPM). The patients/participants provided their written informed consent to participate in this study.

## Author Contributions

All authors listed have made a substantial, direct and intellectual contribution to the work, and approved it for publication.

## Conflict of Interest

The authors declare that the research was conducted in the absence of any commercial or financial relationships that could be construed as a potential conflict of interest.
